# Unraveling the hidden complexity of cancer through long-read sequencing

**DOI:** 10.1101/gr.280041.124

**Published:** 2025-04

**Authors:** Qiuhui Li, Ayse G. Keskus, Justin Wagner, Michal B. Izydorczyk, Winston Timp, Fritz J. Sedlazeck, Alison P. Klein, Justin M. Zook, Mikhail Kolmogorov, Michael C. Schatz

**Affiliations:** 1 Department of Computer Science, Johns Hopkins University, Baltimore, Maryland 21218, USA;; 2 Cancer Data Science Laboratory, Center for Cancer Research, National Cancer Institute, Bethesda, Maryland 20892, USA;; 3 Material Measurement Laboratory, National Institute of Standards and Technology, Gaithersburg, Maryland 20899, USA;; 4 Human Genome Sequencing Center, Baylor College of Medicine, Houston, Texas 77030, USA;; 5 Department of Biomedical Engineering, Johns Hopkins University, Baltimore, Maryland 21218, USA;; 6 Department of Molecular and Human Genetics, Baylor College of Medicine, Texas 77030, USA;; 7 Department of Computer Science, Rice University, Houston, Texas 77251, USA;; 8 Sidney Kimmel Comprehensive Cancer Center, Department of Oncology, Johns Hopkins Medicine, Baltimore, Maryland 21031, USA

## Abstract

Cancer is fundamentally a disease of the genome, characterized by extensive genomic, transcriptomic, and epigenomic alterations. Most current studies predominantly use short-read sequencing, gene panels, or microarrays to explore these alterations; however, these technologies can systematically miss or misrepresent certain types of alterations, especially structural variants, complex rearrangements, and alterations within repetitive regions. Long-read sequencing is rapidly emerging as a transformative technology for cancer research by providing a comprehensive view across the genome, transcriptome, and epigenome, including the ability to detect alterations that previous technologies have overlooked. In this Perspective, we explore the current applications of long-read sequencing for both germline and somatic cancer analysis. We provide an overview of the computational methodologies tailored to long-read data and highlight key discoveries and resources within cancer genomics that were previously inaccessible with prior technologies. We also address future opportunities and persistent challenges, including the experimental and computational requirements needed to scale to larger sample sizes, the hurdles in sequencing and analyzing complex cancer genomes, and opportunities for leveraging machine learning and artificial intelligence technologies for cancer informatics. We further discuss how the telomere-to-telomere genome and the emerging human pangenome could enhance the resolution of cancer genome analysis, potentially revolutionizing early detection and disease monitoring in patients. Finally, we outline strategies for transitioning long-read sequencing from research applications to routine clinical practice.

A hallmark of cancer is widespread genetic and epigenetic instability (Hanahan and Weinberg 2011). Cancer often originates through somatic mutations that accumulate throughout an individual's lifetime due to exposure to carcinogens, DNA replication errors, and other factors, thereby leading to the clonal evolution of cancer cells (Qing et al. 2020; Chakravarty and Solit 2021). Somatic mutations in key genes and their regulatory sequences, especially oncogenes, tumor suppressors, and DNA repair genes, are frequently found in cancers and contribute to tumor progression and therapeutic resistance (Olivier et al. 2010; Prior et al. 2012). In addition, germline pathogenic variants, while less prevalent than somatic mutations for driving cancer risk, account for at least 5%–10% of all cancers. These inherited variants originate in reproductive cells and are passed from parents to offspring (Pudjihartono et al. 2022). Clinically pathogenic variants, particularly those in high-penetrance genes such as *BRCA1/2* and *APC*, are of primary importance in cancer risk assessment and treatment (Preisler et al. 2021; Nepomuceno et al. 2024).

The rapid advancement of genome sequencing technologies over the past 30 years has revolutionized our ability to catalog cancer risk variants and understand the genomic landscape of cancer. Early studies relied on targeted sequencing and microarrays to detect genomic and transcriptomic variations enriched in cancer patients, revealing, for example, the prevalence of widespread mutations in *TP53* (Nigro et al. 1989) and the molecular basis of the subtypes of breast cancers (Perou et al. 2000). While exome-wide studies were possible with these technologies, sample sizes were modest (Wood et al. 2007; Jones et al. 2009). Nevertheless, these technologies remain clinically important to this day due to their predictable performance and low costs.

More recently, short-read sequencing has gained widespread adoption for cancer research as it enables the genome-wide identification of alterations, including single-nucleotide variants (SNVs), small insertions and deletions (indels), copy number alterations, and some structural variants (SVs) (Choo et al. 2023). This high-throughput, cost-effective technology has facilitated large-scale cancer genomics projects like The Cancer Genome Atlas (TCGA) (The Cancer Genome Atlas Research Network et al. 2013), the International Cancer Genome Consortium (ICGC) (Zhang et al. 2019), and the Pan-Cancer Analysis of Whole Genomes (PCAWG) project (The ICGC/TCGA Pan-Cancer Analysis of Whole Genomes Consortium 2020) to characterize thousands of cancers across dozens of cancer types. Building on these population-scale efforts, the Catalogue Of Somatic Mutations In Cancer (COSMIC) has emerged as a comprehensive resource for somatic mutation data (Tate et al. 2019). In its latest release, COSMIC V100 expanded to include over 24 million gene mutations, incorporating genome-wide sequencing results from tens of thousands of tumors across diverse cancer types. These collaborative efforts have yielded invaluable insights into the molecular mechanisms underlying cancer initiation, progression, and metastasis, uncovering recurring patterns of genomic alterations, novel cancer genes, and pathways. Consequently, short-read sequencing for both tumor and germline is routinely conducted as part of patient management.

While short-read sequencing has greatly advanced our understanding of cancer susceptibility and progression, it faces several major challenges that have systematically excluded certain parts of the genome and certain types of variations from studies. Most notably, short-read sequencing is notoriously limited for germline analysis of structural variations, defined as any variant at least 50 bp in size, including insertions, deletions, inversions, duplications, and other complex variant types. While fewer in number than SNVs, because of their larger size, SVs account for a larger number of variant bases across the germline genome. SVs are also often strongly associated with alterations in gene expression (Chiang et al. 2017; Scott et al. 2021), and are emerging as an important source of pathogenic variations (Kleinert and Kircher 2022). In addition to SVs, short-reads struggle with detecting alterations in tandem repeats (TRs), and more generally, with variations found within repetitive sequences (e.g., segmental duplications, satellite sequences, transposable elements), leaving a large fraction of the genome inaccessible. Short-reads also have limited power to phase variants within the same haplotype, meaning they often cannot conclusively determine if there has been a complete loss of function in genes from compound heterozygous mutations (Sedlazeck et al. 2018). In addition to these limitations for detecting DNA variations and mutations, short-reads also face related challenges resolving alterations in transcriptomes or epigenomes and cannot simultaneously capture genomic and epigenomic variations, which further hinders the phasing of methylation and the detection of allele-specific methylation (ASM).

Addressing these challenges, long-read sequencing technologies, such as Pacific Biosciences (PacBio) Single Molecule Real Time sequencing and Oxford Nanopore Technologies (ONT) sequencing, have emerged as powerful alternatives to short-read sequencing (Fig. 1; van Dijk et al. 2023). Their extended read lengths can span difficult-to-resolve regions, improving the identification performance of structural variants, as well as improving the identification and phasing of variations in repetitive regions (Fig. 1B). The growth in adoption has mirrored the overall improvements to these platforms: while the initial release of these technologies was plagued by high error rates, high costs, and low throughput, current long-read sequencing platforms have achieved high accuracy and throughput at competitive costs (Kovaka et al. 2023) promoting a variety of new applications.

**Figure 1.**
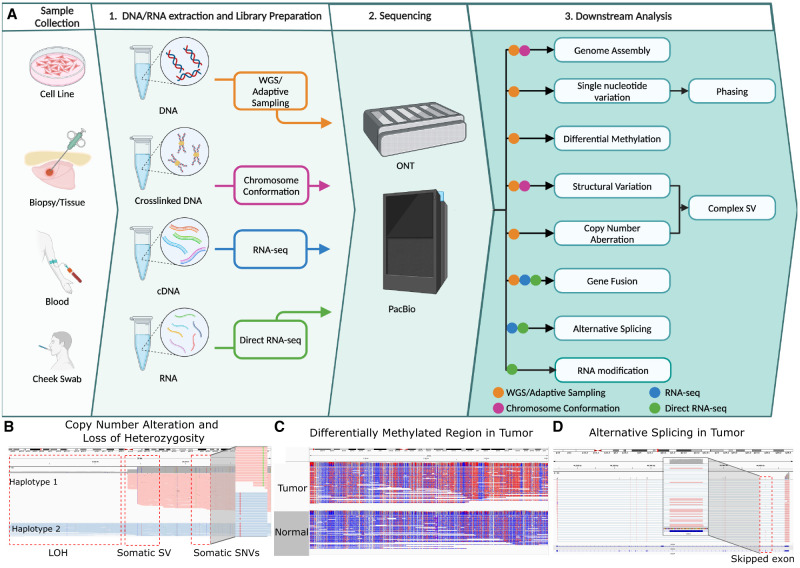
Overview of long-read sequencing protocols and analyses. (*A*) The overview of the long-read sequencing workflow including downstream analysis options. (*B*) Somatic SNV and a structural variation in the COLO829 cell line resulting in haplotype-specific copy number change and loss of heterozygosity (data from Keskus et al. 2024). Reads are grouped and colored with the alleles using long-read-based phasing. (*C*) Differentially methylated region between tumor and normal sample. Reads are colored with 5mC modifications (red: high methylation, blue: low methylation). (*D*) An alternative splicing in *CASC4* gene in cancer cell line SCC152 (Rodriguez et al. 2024).

One of the most notable examples of the performance of long reads was how in 2022 long-read sequencing was used to produce the first telomere-to-telomere (T2T) assembly of a human genome (Nurk et al. 2022). Compared to previous references, the new T2T reference genome corrected thousands of structural errors and revealed >100 Mbp of previously uncharacterized regions of the human genome. Moreover, long reads have been essential to develop population-scale references of structural variations (SVs) in the human population (Ebert et al. 2021; Liao et al. 2023; Mahmoud et al. 2024), as well as to detect novel gene models and epigenetic changes (Gershman et al. 2022; Glinos et al. 2022; Kolmogorov et al. 2023; Kovaka et al. 2024; Stefansson et al. 2024). In addition to its transformative impact on basic sciences, long-read sequencing is emerging as a critical tool in clinical diagnostics. Long-read's ability to accurately detect SVs and other complex variants missed by traditional short-read sequencing has proven invaluable in identifying causal SVs in Mendelian disease (Merker et al. 2018), repeat expansions underlying neurodevelopmental disorders (Ishiura et al. 2018; Hiatt et al. 2021), and many other conditions (Marwaha et al. 2022; Damaraju et al. 2024). A particularly notable clinical application of long-read sequencing is the development of rapid (same day) sequencing workflows (Gorzynski et al. 2022), which are crucial for critically ill patients, where timely and accurate genetic diagnosis can directly influence treatment decisions, potentially improving prognosis and reducing healthcare costs.

Within cancer genomics, long-read sequencing has similarly witnessed growing adoption, especially to resolve SVs and other complex variations (Sakamoto et al. 2020a, 2021b). The earliest applications used long-read sequencing of cancer cell lines to validate known structural variations (Norris et al. 2016) and then construct whole-genome catalogs of variations for the first time (Nattestad et al. 2018). These early studies revealed that these cell lines contained tens of thousands of structural variations, most of which had been missed by short-read sequencing, as well as intricate genomic rearrangements in key oncogenes and novel gene fusions (Nattestad et al. 2018). More recent studies have expanded the scope of this work to consider larger numbers of patient samples and more complex types of variation, as well as transcriptomic and epigenomic readouts (Fig. 1B–D; Sereewattanawoot et al. 2018; Aganezov et al. 2020; Sakamoto et al. 2020b, 2022; Zheng et al. 2024a). These efforts have consistently demonstrated the superior capability of long-read sequencing technologies in constructing a more comprehensive cancer genome landscape and elucidating their functional consequences. Here, we summarize the rise of these efforts, focusing on the unique opportunities long reads offer for cancer analysis across the genome, transcriptome, and epigenome (Fig. 1A–D). We then discuss the remaining needs and challenges, and conclude with our perspective on the path toward integrating long reads as a routine component of clinical cancer diagnosis and treatment.

## Long-read genome sequencing

### Growth of germline population sequencing and resources

Before advancing to cancer genomes, long-read sequencing was initially applied to improve the resolution of the reference human genome and to develop comprehensive catalogs of human genetic variation in healthy donors. The first genome-wide analysis of a human genome using long reads was published by Chaisson et al. (2015). In this work, they sequenced a haploid human cell line (CHM1) with PacBio SMRT technology, and found using these data they could close or extend 55% of the remaining gaps in the GRCh37 reference genome—78% of which contained extensive degenerate short TRs, particularly in (G + C)-rich areas. They also resolved over 26,000 structural variants at base pair resolution, including previously undetectable inversions, complex insertions, and long TR tracts. This landmark study highlighted the increased complexity of the human genome, particularly in repetitive DNA regions, which could now be more completely and accurately resolved with long-read sequencing.

In the decade following, further advances in generating longer, more accurate reads enabled the T2T consortium to achieve the first complete human genome sequencing. This began in 2020 with the publication of the first gapless assembly of the human X Chromosome using ultra-long-read nanopore sequencing (Miga et al. 2020). Then in 2022, the consortium published T2T-CHM13 as the first fully gapless human genome assembled primarily from PacBio HiFi and ONT Ultralong reads. This genome comprises 3.055 billion base pairs and addresses the previously unresolved 8% of the genome, including complex heterochromatic regions (Nurk et al. 2022). In 2023, the consortium finalized the complete Y Chromosome sequence (T2T-Y) from the HG002 donor (Rhie et al. 2023). The integration of T2T-Y with the CHM13 reference, along with population variation, clinical variants, and functional genomics data, has created a comprehensive reference for all 24 human chromosomes. The use of the T2T-CHM13 genome as a reference broadly improves our ability to detect variation in human genomes. Specifically, T2T-CHM13 improves the Mendelian concordance rate among trios and eliminates tens of thousands of spurious SNVs per sample, including a reduction of false positives in 269 challenging, medically relevant genes by up to a factor of 12 (Aganezov et al. 2022). Altogether, the T2T consortium has opened previously inaccessible genomic regions, such as centromeric satellite arrays and recent segmental duplications, to variation and functional studies, bringing the routine completion of the entire human genome within reach.

Beyond establishing reference genomes, long reads have been used to probe the genetic landscape of diverse populations and enable the comprehensive characterization of genetic variation through fully reconstructed haplotypes. For example, Seo et al. (2016) used PacBio single-molecule real-time sequencing to construct a contiguous diploid human genome assembly from a Korean individual (AK1), uncovering thousands of previously unreported Asian-specific structural variants and providing high-quality haplotype information of clinically relevant alleles. More recently, in 2021, long reads from PacBio were used to analyze a diverse human panel representing 25 globally diverse populations, uncovering 15.8 million SNVs, 2.3 million indels, and over 107,000 SVs, 42% of which were previously unidentified, particularly in African genomes (Ebert et al. 2021). Following this and related efforts, in 2023 the Human Pangenome Reference Consortium introduced a first draft of the human pangenome reference, which includes 47 phased, diploid assemblies from a genetically diverse cohort (Liao et al. 2023). These assemblies cover over 99% of the expected sequence in each genome and are more than 99% accurate at both the structural and base pair levels. This pangenome adds 119 million base pairs of euchromatic polymorphic sequences and 1115 gene duplications relative to the existing GRCh38 reference, with ∼90 million of these additional base pairs originating from SVs. Furthermore, using the pangenome for analyzing short-read data reduced small variant discovery errors by 34% and increased the detection of SVs per haplotype by 104% compared to GRCh38-based workflows.

Current applications of long-read sequencing technologies are enabling even larger population studies, enhancing our ability to associate genetic variations—particularly those previously undetectable—with human diversity, disease, and other phenotypes. For example, in 2021, the deCODE genetics initiative generated a major large-scale long-read SV callset using Oxford Nanopore sequencing from 3622 Icelanders, identifying three to five times more SVs per sample than short-read data (Beyter et al. 2021). This study also uncovered SVs in strong linkage disequilibrium with disease- or trait-associated variants from the genome-wide association study (GWAS) catalog. This study effectively doubled the number of detected variants compared to short-read sequencing alone. Subsequent efforts have extended to additional diverse populations, such as the resequencing of 1000 Genomes Project samples using long reads that discovered extensive SVs missed in earlier studies (Gustafson et al. 2024; Schloissnig et al. 2024). One of the largest long-read projects to date is the *All of Us* Research Program, which has employed PacBio HiFi reads to sequence over 1000 African American samples in the phase I project (Mahmoud et al. 2024) and is progressing to over 10,000 samples in phase II to be completed by 2025. Meanwhile, the Consortium of Long Read Sequencing (CoLoRS, https://colorsdb.org/) has established an open-resource for population-wide variation cataloging. By aggregating nearly 1400 long-read genomes from various institutions, CoLoRS provides a comprehensive data set with diverse characteristics in read depth, disease focus, trio availability, and ancestry, facilitating the exploration of genetic diversity across different populations and disease states. Additionally, the NIH Center for Alzheimer's and Related Dementias (CARD) has implemented nanopore sequencing technology to analyze brain samples and cell lines (Kolmogorov et al. 2023). CARD intends to expand its analysis to thousands of samples, aiming to yield unprecedented insights into the genetic foundations of Alzheimer's and related dementias.

### Benchmarking standards for germline analysis

**Figure 2.**
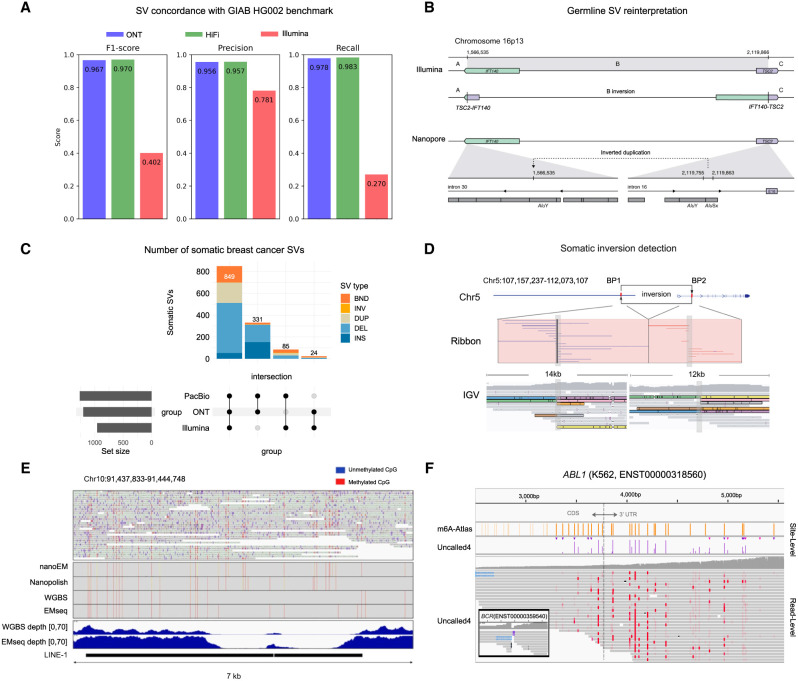
Comparative analysis of structural variation and epigenetics using short- and long-read sequencing. (*A*) Performance comparison of structural variant detection across sequencing technologies, evaluated against the GIAB Tier1 V0.6 benchmark (Kolmogorov et al. 2023). (*B*) Reinterpretation of a germline recurrent event using long-read sequencing. (*Upper* panel) Illumina data indicate a long-range inversion on Chromosome 16p13 with breakpoints in *IFT140* and *TSC2*. (*Lower* panel) ONT sequencing reveals the actual structure: an insertion in intron 30 of *IFT140* (Thibodeau et al. 2020). (*C*) UpSet plot of somatic structural variants identified in the breast cancer cell line using three sequencing technologies (Keskus et al. 2024). (DEL) Deletion, (BND) breakend junction, (DUP) duplication, (INV) inversion, (INS) insertion. (*D*) 4.9 Mbp somatic inversion detected by nanopore sequencing in colorectal cancer, encompassing exon 1 of the *APC* gene (Xu et al. 2023). (*E*) Methylation patterns of LINE-1 located in *HECTD2* intron. (*Upper* panel) Reads from breast cancer cell lines aligned to the region. (*Lower* panel) Read coverage of EMseq and WGBS for the same region (Sakamoto et al. 2021a). (*F*) m6A profiling of an *ABL1* transcript and *BCR-ABL1* fusion in chronic myeloid leukemia (CML) (Kovaka et al. 2024). (C–F, adapted from Thibodeau et al. 2020, Sakamoto et al. 2021a, Xu et al. 2023, and Kovaka et al. 2024).

In parallel to population-wide sequencing efforts, the Genome in a Bottle Consortium (GIAB), the Platinum Genomes Project (Eberle et al. 2017), and related efforts, have developed benchmarks for germline variant calling using a small number of extensively characterized normal cell lines. The first GIAB benchmark from 2014 included ∼77% of autosomal bases in GRCh37 (Zook et al. 2014). However, this excluded challenging regions and variants, including medically relevant regions (e.g., one large study found that one in seven pathogenic variants was challenging to detect with short-reads) (Lincoln et al. 2021). Therefore, GIAB has expanded benchmarks to include increasingly difficult regions and variants, such as SVs (Zook et al. 2020), TRs (English et al. 2024), the Major Histocompatibility Complex (Chin et al. 2020), challenging medically relevant genes including those identified from COSMIC (Wagner et al. 2022), and the X and Y Chromosomes (Wagner et al. 2025). The latest draft benchmarks currently being evaluated by GIAB are based on a T2T assembly of HG002 and include 3.6 million SNVs, 950,000 indels, and 29,000 SVs in 2.74 and 2.76 Gbp of GRCh38 (2.77 and 2.82 Gbp of CHM13) for small variants and SVs, respectively (https://github.com/marbl/HG002). Long-read sequencing has demonstrated superior performance in SV detection compared to Illumina-based approaches (Fig. 2A; Kolmogorov et al. 2023). Nevertheless, the T2T assembly of CHM13v2.0 with X and Y Chromosomes is >3.1 Gbp, so substantial regions are still excluded from variant benchmarks even with near-perfect assemblies due to challenges in aligning, representing, and comparing large complex SVs around segmental duplications and satellite repeats.

Related studies such as the PrecisionFDA Challenge (Olson et al. 2022) helped show how long reads improved variant call accuracy in regions difficult to map with short-reads, though short-reads still had advantages in certain contexts like homopolymers. This has led to a virtuous cycle where benchmarks drive advancements in sequencing technologies and bioinformatics methods, and method improvements, in turn, enable expanded benchmarks. In parallel, genome stratifications help determine regions where variants are more likely identified through long-read approaches, such as 12 genes from the COSMIC Cancer Gene Census are entirely located within a segmental duplication on GRCh38 (Wagner et al. 2022; Dwarshuis et al. 2024).

### Germline variation analysis in cancer patients

Germline variants influence cancer susceptibility by modifying the regulation or function of essential genes and pathways. Every human carries millions of variations in their germline compared to the reference genome, including hundreds to thousands of rare and common variants that affect disease and cancer risks (MacArthur et al. 2012). Consequently, there is interest to use long-read sequencing to explore SNVs, indels, SVs, repeat expansions, and other complex variations that were previously missed or incorrectly identified in an effort to identify new risk factors for disease. These efforts also help establish the landscape of germline variation to aid in the interpretation of somatic variation, as we discuss below.

### The impact of long-read sequencing on variant detection and clinical interpretation

In one of the first studies using long-read sequencing of patient germline genomes, Aganezov et al. (2020) used multiple short- and long-read sequencing technologies to analyze the germline genomes from breast cancer patients. The study demonstrated that long-read sequencing allows for substantially more accurate and sensitive SV detection, achieving 90%–95% concordance between the PacBio and ONT long-read platforms, including in both the germline and cancer genomes. SVs detected exclusively with short-reads that occur near different types of long-read variants or within TR regions were often found to be miscalls from short-reads. Notably, the researchers identified hundreds of variants in two cancer patients’ germline genomes within known cancer-related genes detectable only through long-read sequencing, including an intronic SV in *BRCA1* that was not detectable using short-reads. Other germline variations in *BRCA1* can increase the lifetime risk of developing breast cancers to more than 85% although it remains unknown if these specific variations carry disease risk. Nevertheless, these and related findings highlight the strong potential of long-read sequencing in cancer genomics for accurately assessing genetic instability.

Long-read sequencing also facilitates the detailed characterization of diverse variants of cancer susceptibility genes, including private, recurrent, and founder variants. For example, a breast cancer study using nanopore sequencing precisely profiled fourteen variants, ranging from single-exon alterations to whole-gene changes (Dixon et al. 2023). They confirmed a 6126 bp tandem duplication in three samples, and reported a shared 1.08 Mb haplotype block by analyzing SNVs beyond the *BRCA1* founder variant boundaries. The accurate identification and phasing of SV breakpoints uncovered previously unrecognized allelic heterogeneity in key genes such as *BRCA1* and *CHEK2* and elucidated the formation mechanisms of recurrent deletions. To achieve more cost-effective identification of disease-causing variations, Nakamura et al. (2024) developed a computational workflow for target adaptive sampling long-read sequencing (see below for a discussion of adaptive sampling) and applied it to 33 suspected hereditary cancer patients. They identified 14 putative pathogenic SNVs and indels, uncovered newly identified SINE-R/VNTR/*Alu* elements affecting the *APC* gene in two familial adenomatous polyposis patients, and demonstrated the utility of off-target reads from adaptive sampling for SNP genotyping, enabling polygenic risk score calculations.

The application of long-read sequencing further reduces false-positive calls and enhances clinical interpretation. A study using nanopore sequencing reassessed SVs initially identified by Illumina in 669 cancer patients, confirming eight pathogenic or likely pathogenic SVs and resolving three additional variants that were ambiguous with short-read data (Thibodeau et al. 2020). For example, a recurrent inversion on Chromosome 16p13, initially misclassified as pathogenic, was revealed to be an inverted duplication of an *Alu* element, leading to its reclassification as likely benign (Fig. 2B). Similarly, Ban et al. (2024) combined long-read sequencing with transcript analysis to identify a novel intragenic duplication in the *PALB2* gene in a patient with triple-negative breast cancer. This *PALB2* duplication was reclassified as pathogenic, suggesting a potential causal link between this genetic alteration and the aggressive phenotype observed in the patient. Further investigation of the tandem duplication mechanism revealed microhomology regions at both proximal and distal breakpoints within *Alu* elements, which were codirectionally aligned with the transcriptional direction of *PALB2*, suggesting the role of repetitive elements in facilitating the duplication event. These advancements demonstrate the potential of long-read sequencing in resolving ambiguous variants and correcting variant classification, thereby improving the cancer screening accuracy.

Moreover, family-based studies using long-read sequencing have advanced our understanding of inherited variations in cancer. Kramer et al. (2024) performed long-read whole-genome sequencing with ONT PromethION on three families with early onset cancer probands, including two colorectal cancer trios and a quad with two siblings affected by testicular cancer, all with unaffected parents. They identified SNVs, SVs, and epigenetic profiles for each individual and applied a family-based filtering strategy to prioritize candidate variants. This approach effectively eliminated nonrelevant variants shared with unaffected parents, facilitating the identification of candidate pathogenic variants, including de novo and compound heterozygous variants (Kramer et al. 2024). In another investigation involving a family with two siblings affected by congenital atypical teratoid rhabdoid tumor, PacBio HiFi sequencing determined the position of an insertion within intron 2 of *SMARCB1*, and identified it to be a SINE–VNTR–*Alu* (SVA) retrotransposon element present in a mosaic state in the mother (Sabatella et al. 2021). A broader analysis of 120 previously unsolved families severely affected by breast, ovarian, pancreatic, or metastatic prostate cancer identified rare deep intronic variants in eight families (6%), where variants in *BRCA1*, *PALB2*, and *ATM* created intronic pseudoexons that were spliced into transcripts, resulting in premature truncations (Gulsuner et al. 2024). Overall, the elucidation of the inheritance patterns of previously hidden variations promises to enhance genetic counseling and surveillance strategies for affected families.

### Advances in variant bioinformatics tools: from detection to interpretation

SNVs and indels are common genetic variants contributing to genetic diversity and disease susceptibility. While short-read methods effectively detect these variants in “high-confidence” regions, they struggle to resolve variants in complex or repetitive genomic regions (Kosugi and Terao 2024). To address these gaps, several tools using long reads have been developed. DeepVariant pioneered small variant calling using convolutional neural networks (Poplin et al. 2018). ClairS implements a deep learning model that uses summaries of adjacent genomic positions around candidate sites (Luo et al. 2020). Later, NanoCaller was developed to improve variant calling by incorporating long-range haplotype information and generating features from distant heterozygous SNPs (Ahsan et al. 2021). PEPPER-Margin-DeepVariant, a haplotype-aware genotyping pipeline, has demonstrated superior performance in identifying small variants from nanopore and PacBio HiFi data, particularly in segmental duplications and low-mappability regions where short-read methods often fail (Shafin et al. 2021). Beyond SNVs and indels, TR detection promotes the further understanding of cancer mechanisms. Straglr performs genome-wide screening for TR expansions by identifying insertions composed of repeat elements and genotyping the expanded loci (Readman et al. 2021). TRcaller enables the characterization of TR alleles from both short- and long-read sequences, achieving high accuracy and sensitivity (Wang et al. 2023).

Multiple studies have shown that long-read sequencing detects two to three times more germline SVs than short-read methods, especially for insertions where long reads capture up to 80% more variants than short-reads (Chaisson et al. 2019; Aganezov et al. 2020; Schloissnig et al. 2024). Many of these newly identified SVs are located in highly repetitive regions associated with various diseases, such as repeat expansions in medically important genes and structural variations affecting key pharmacogenes (Gustafson et al. 2024). Therefore, long-read sequencing has become instrumental in comprehensive SV detection, with algorithms broadly categorized into alignment-based and assembly-based methods. Alignment-based tools, such as Sniffles2 (Smolka et al. 2024), cuteSV (Jiang et al. 2020), and SVIM (Heller and Vingron 2019), analyze reads mapped to a reference genome, identify SV signatures, and aggregate similar signatures to produce consensus SV calls. While computationally faster than assembly-based methods, they struggle with diverse SV signatures for complex variants as well as sequencing and alignment errors. Recent advancements have achieved more precise SV profiling, particularly for large inversions and translocations in cancer genomes (Smolka et al. 2024).

Assembly-based methods reconstruct genomic sequences into longer contigs before comparing them with a reference genome. Assembly-based methods are further divided into de novo and reference-guided local assembly approaches. De novo assembly tools like phased assembly variant (PAV) (Ebert et al. 2021) and SVIM-asm (Heller and Vingron 2021) excel at detecting large-scale alterations and novel insertions. But these approaches can miss heterozygous variants if they are not resolved in the assembly and require computationally intensive genome-wide assembly before variant identification. Furthermore, assembly-based methods are often unable to capture mosaic mutations that are often prevalent in cancer tissues as they may have insufficient coverage to find these mutations. Comparatively, reference-guided local assembly methods provide effective SV identification and mitigate issues such as assembly collapse around segmental duplications. For example, DeBreak employs a local de novo assembly approach to cluster SVs and reconstruct long insertions, and it offers a specialized “tumor” mode for analyzing cancer genomes (Chen et al. 2023a).

The increasing cost-effectiveness of long-read sequencing has enabled large-scale cancer genomics studies, facilitating the construction of cohort-level SV callsets to classify rare and common variants potentially implicated in cancer predisposition or progression. Relatedly, despite major advancements in SV detection methods for individual samples, comprehensively profiling all SV types in a sample often requires integrating results from multiple bioinformatics algorithms. To address the challenges, several specialized SV merging tools have been developed to improve the analysis of complex genomic structures at both individual and population levels. Early methods integrate structural variants based on their coordinates and user-defined parameters such as size and type (Jeffares et al. 2017). Recently developed tools have further improved the SV merging performance by considering both sequence similarity and genomic position (English et al. 2022; Kirsche et al. 2023; Zheng et al. 2024b). These approaches aim to maintain high genotype accuracy and optimize computational efficiency at both individual and population scales. With the established cohort-level variant callset/pangenome, variants in an individual of interest can be characterized through genotyping, i.e., determining an individual's genotype for each known variant in the data set (Chen et al. 2019; Ebler et al. 2022; Grytten et al. 2023). Notably, genotyping can be performed within both short-read and long-read data sets, allowing researchers to tap into the large numbers of patient genomes sequenced with short-reads, albeit with reduced performance in repetitive sequences. Nevertheless, the genotyping process not only enhances the sensitivity and specificity of variant calling but also serves as a critical step in population genetics, quantitative trait locus mapping, and genome-wide association studies.

The related problem of the interpretation of variants and their impact on phenotypes and diseases has gained increasing attention in recent years. For small variants, several tools were developed to assess pathogenicity, especially for missense variants and small in-frame indels. The Combined Annotation Dependent Depletion (CADD) method offers a standardized, genome-wide scoring system (C-score) by integrating sequence conservation and functional annotations (Kircher et al. 2014). FATHMM-MKL improves predictions of functional consequences for both coding and noncoding variants by optimally weighting diverse genomic features (Shihab et al. 2015). To streamline the annotation process, PhD-SNPg, a lightweight, sequence-based machine learning tool, enables efficient analysis of SNPs and indels across coding and noncoding regions (Capriotti and Fariselli 2023). With advancements in SV detection, tools like SVScore (Ganel et al. 2017) and AnnotSV (Geoffroy et al. 2018), integrate multiple genomic features to annotate SVs and identify potentially deleterious ones. Later, Kumar et al. (2020) developed SVFX, a machine learning-based workflow that quantifies the pathogenicity of both somatic and germline SVs. Within cancer cohorts, the predicted pathogenic SVs were enriched in known cancer genes and pathways (Kumar et al. 2020). CADD-SV enhances the SV effect prediction by integrating diverse variant annotations and employing random forest models, producing scores that correlate to known pathogenic and rare variants (Kleinert and Kircher 2022).

Relatedly, the availability of population-level SV data sets has enabled more accurate stratification of variants as common or rare in the general population, thereby improving SV annotation performance (Nicholas et al. 2022). This has led to the development of methods for analyzing rare variations. For example, a recent advancement, Watershed-SV, employs a probabilistic model integrating diverse omic signals and novel SV-related features to characterize the functional effects of rare SVs on expression (Jensen et al. 2025). When applied to the Undiagnosed Diseases Network patient data set, Watershed-SV identified more disease-relevant rare SV candidates than CADD-SV, offering valuable insights into gene disruption mechanisms. These evolving tools promote a more comprehensive and accurate evaluation of variant effects. While these tools help to identify candidate variants, functional studies are still needed before these findings can directly impact diagnostic and therapeutic strategies in clinical genomics.

### Identification, analysis, and benchmarking of somatic variations

Accurate and complete detection of somatic variation is essential for cancer genomics studies aiming to identify and catalog driver mutations (Cortés-Ciriano et al. 2021), study tumor evolution (Gerstung et al. 2020), stratify cancers into clinical subtypes (Chakravarty and Solit 2021), and inform patient treatment (Sosinsky et al. 2024). Somatic variation in cancer is a result of various mutational processes (Carvalho and Lupski 2016) that could be specific to cancer types or environmental exposures (Alexandrov et al. 2020). In addition to gradually accumulating somatic mutations, cancer genomes are often characterized by genomic instability (Drews et al. 2022) and rearranged genomes (Li et al. 2020), which facilitate rapid tumor evolution (Stephens et al. 2011). As long-read sequencing continues to advance cancer genomics, it promises to unveil more complex genetic mechanisms underlying cancer predisposition and to deepen our understanding of the interplay between germline variants and somatic mutations across various cancer types.

### Early studies overcoming challenges of long-read sequencing of tumors

Tumor clonality, imperfect purity (i.e., normal cells in tumor sample), tumor in normal (TIN) contamination, and loss of heterozygosity events complicate the interpretation of sequencing data, and generally require deeper sequencing to capture low-abundance variants. Extracting high molecular weight (HMW) DNA from tumor tissues (rather than cell lines or blood) is often challenging due to lower input material volumes. Either fresh or high-quality fresh frozen samples are currently required to produce long-read DNA libraries, which is a limitation as most archival tumor samples are formalin-fixed paraffin-embedded (FFPE). To distinguish between germline and somatic variants, tumor samples are often complemented by matching normal samples (The ICGA/TCGA Pan-Cancer Analysis of Whole Genomes Consortium 2020). This is highly effective in identifying somatic variants as those not observed in the paired normal samples, although it suffers from increased sequencing costs. An alternative strategy is to use panels of normals that represent the variation commonly observed in a population; such panels are produced by the population studies highlighted above. This strategy is effective for filtering common variations, although it can lead to misclassification of rare variants and variants in challenging regions that are not captured in the panel of normals.

Initial studies applied long-read sequencing to cancer cell lines and organoids. One early study from 2016 (Norris et al. 2016) used ONT sequencing to detect well-characterized SVs, including large deletions, inversions, and translocations in pancreatic cell lines. Another study from 2018 (Nattestad et al. 2018) performed PacBio sequencing to detect germline and somatic SVs in the SKBR3 breast cancer cell line, revealing highly complex rearrangements around the *ERBB2* oncogene and extensive rearrangements and other SVs genomewide. In a related study from 2020 (Aganezov et al. 2020), the authors showed high consistency and specificity of PacBio and ONT technologies for SV discovery in breast cancer patients-derived organoids.

More recent studies demonstrated the feasibility of producing long-read sequencing from various clinical tumor samples. For example, Sakamoto et al. (2020b) characterized regions of clustered copy number changes, inversions, and deletions in 20 lung adenocarcinoma patients. This team further used long-read phasing to illustrate the uneven distribution of somatic mutations between haplotypes (Sakamoto et al. 2022). In another study, Fujimoto et al. (2021) analyzed mechanisms of somatic insertions in 11 clinical liver samples with matching normals. Recently, ONT sequencing was applied to 189 advanced tumors from the Personalized OncoGenomics (POG) program, demonstrating the potential of long-read sequencing as a scalable precision oncology tool (O'Neill et al. 2024).

### Advantages of long-read sequencing for somatic variation detection

Compared to germline SVs, a higher proportion of somatic SVs are accessible to short-read sequencing because somatic SV breakpoints are less likely to coincide with repetitive elements (Carvalho and Lupski 2016). Indeed, a recent study highlighted that certain classes of somatic SVs (such as large copy number alterations) can be reliably detected with short-reads without the need for long reads (Choo et al. 2023). However, their analysis focused exclusively on copy number alternations larger than 10 kb, which represent only a small fraction of SVs (roughly 10% or less), limiting the applicability of their comparison between short- and long-read technologies. Other studies (Keskus et al. 2024) demonstrated that short-read methods have “blind spots” and miss certain SV types, such as clustered SVs or insertions (Fig. 2C; Mahmoud et al. 2019). In addition, short-read somatic SV calls do not always achieve sequence-level resolution of breakpoints and insertions and typically produce more false-positive calls (Keskus et al. 2024), which might impact the interpretation (Mahmoud et al. 2019). For example, Xu et al. (2023) identified a 4.9 Mbp inversion in a colorectal cancer sample (C546-T) that encompassed exon 1 of the *APC* gene (Fig. 2D). RNA-seq analysis revealed a substantial decrease in *APC* expression in the tumor sample (tumor fragments per kilobase of transcript per million fragments mapped (FPKM): 0.296 vs. normal FPKM: 2.262). The SV was not detected by short-read sequencing, likely because the base sequence of the inverted exon remained unchanged. Since most published cancer genomics studies are based on short-reads, our current understanding of the landscape of somatic SVs in cancer is likely to be incomplete.

Long-read sequencing has enabled the analysis of difficult-to-map repetitive DNA, which can play an important role in cancer progression (Erwin et al. 2023). For example, centromeres and telomeres play a key role in maintaining genome stability and are involved in cancer mutational processes such as breakage-fusion-bridge (BFB) amplification (Gisselsson et al. 2000). A recent study characterized various classes of rearrangements involving telomeric repeats using long-read sequencing, including neotelomeres and chromosome arm fusions (Tan et al. 2024). New targeted long-read-based technologies are also being developed to profile changes in telomere lengths (Schmidt et al. 2024).

Some cancers could be driven by viral integrations or LINE-1 retrotranspositions that are difficult to fully resolve using short-reads and, therefore, will benefit from long-read sequencing (Rodriguez-Martin et al. 2020). For example, a recent study used long-read sequencing to discover reciprocal chromosomal translocations mediated by L1 activity (Zumalave et al. 2024). Another work profiled integrations of human papillomavirus (HPV) in 16 cervical cancers using ONT sequencing, revealing different types of integrations and SVs around the integration sites (Zhou et al. 2022). Other recent applications of long-read sequencing to HPV-infected cancers revealed complex HPV-host concatemers that exist as extrachromosomal amplicons and drive complex rearrangements and intratumoral heterogeneity (Akagi et al. 2023; Rossi et al. 2023).

Long-read connectivity can also be used to better characterize the structure and mechanisms of complex rearrangement and amplification processes, such as chromothripsis, extrachromosomal DNA (ecDNA), or BFB. For example, one germline study used nanopore sequencing to identify disease-related gene losses resulting from chromothripsis in a Langer–Giedion syndrome (Lei et al. 2020). Using ONT sequencing of a childhood medulloblastoma, Rausch et al. (2023) discovered a new SV type termed templated insertion thread and assembled a chromosome copy affected by chromothripsis. Ng et al. (2024) used long-read de novo assembly to resolve complex BFB and ecDNA amplicons in nine cases of esophageal adenocarcinoma. Rodriguez et al. (2024) analyzed 19 cervical cancer cell lines to reveal recurrent BFB events that amplify *YAP1-BIRC3-BIRC2* genes, associated with 10-year-earlier age of diagnosis and three times more common in African American women.

Finally, liquid biopsy is a promising alternative to traditional tissue biopsies in cancer management, offering a minimally invasive method for analyzing circulating tumor cells, cell-free DNA (cfDNA), and other biomarkers in bodily fluids (Ignatiadis et al. 2021; Zhu et al. 2023). Recent advancements in long-read sequencing technologies have further enhanced liquid biopsy capabilities, allowing for real-time analysis of cfDNA genomic and fragmentomic signatures and the detection of previously unobserved longer cfDNA fragments in cancer patients (van der Pol et al. 2023).

### New algorithms and tools for long-read somatic variation analysis

Analysis of cancer genomes presents additional computational challenges over germline analysis as most of the existing tools for long reads were not optimized to work with matching normal samples, variable tumor clonality, allelic imbalance, loss of heterozygosity, and complex somatic SV patterns. The recently developed deep learning-based method ClairS (Zheng et al. 2023) supports matching normal samples and takes advantage of long-read phasing to improve the detection of variants with low allelic fractions; DeepSomatic (Park et al. 2024) is another recently developed tool that was trained using a panel of multiple cancer cell lines with different mutational patterns (Table 1). For somatic SV analysis, GASOLINE jointly extracts SV signatures from tumor and normal samples to distinguish somatic from germline variants (Magi et al. 2023a). nanomonsv (Shiraishi et al. 2023) uses a local assembly approach to distinguish somatic and germline variants and introduced a single breakend mode to represent SVs that are partially mappable (Table 1). SAVANA (Elrick et al. 2024) employs a machine learning-based classifier as an additional filter for somatic variant calls to improve precision. Sniffles2 (Smolka et al. 2024) implemented a new function for multisample joint SV genotyping that can be used to separate germline and somatic variants. The recently developed Severus (Keskus et al. 2024) algorithm incorporates long-read phasing to produce haplotype-resolved SV calls and uses a breakpoint graph approach to detect complex rearrangements that consist of many clustered SVs (Table 1).

**Table 1.** Summary of software tools for analyzing long reads in cancer

Category	Tool name	Description	Reference
Alignment	NGMLR	Convex gap-cost scoring model to achieve long-read alignment	Sedlazeck et al. 2018
	minimap2	Pairwise alignment method for long reads and large genomes	Li 2018
	Winnowmap	Improvements in aligning long reads in repetitive regions	Jain et al. 2020
Small variant detection	ClairS	Deep learning method designed for detecting somatic small variants, primarily for ONT long-read data	Zheng et al. 2023
	DeepSomatic	Discovery of somatic small variants across multiple sequencing platforms	Park et al. 2024
SV calling	nanomonsv	Identification of somatic SVs at single-nucleotide resolution	Shiraishi et al. 2023
	SAVANA	Somatic SV and copy number aberrations caller for long reads	Elrick et al. 2024
	SVision-pro	Neural network framework enabling somatic structural variant discovery and genotyping	Wang et al. 2024
	Sniffles2	Updated version of Sniffles; capable of identifying mosaic and population-level SVs using long reads	Smolka et al. 2024
	Severus	Improved detection and characterization of somatic SVs in tumor genomes	Keskus et al. 2024
Variant phasing	WhatsHap	Phasing of SNVs and smaller indels	Martin et al. 2023
	LongPhase	Fast chromosome-scale phasing for both small and large variations	Lin et al. 2022
Methylation identification	DeepSignal	Detection of DNA methylation states from ONT long reads	Ni et al. 2019
	Uncalled4	Toolkit for ONT signal alignment, analysis, and visualization, improving DNA and RNA modification detection	Kovaka et al. 2024
	ccsmeth	DNA 5mCpGs caller for PacBio CCS data	Ni et al. 2023
Methylation phasing	NanoMethPhase	Phasing of 5mCpGs from ONT sequencing data	Akbari et al. 2021
	MethPhaser	Utilization of ONT methylation signals to extend SNV-based phasing	Fu et al. 2024
	ccsmethphase	Nextflow pipeline for haplotype-aware methylation detection using PacBio CCS reads	Ni et al. 2023

Long-range phasing of somatic and germline variants is another advantage of long-read technologies, facilitating the discovery of biallelic variants (O'Neill et al. 2024) and improving the accuracy of the somatic/germline variant classification (Simpson 2024). For example, a study on nonsmall cell lung cancer generated 834 kb long-phased blocks and used the phased information to uncover regions with uneven mutation distribution between haplotypes (Sakamoto et al. 2022). A more recent study of long-read sequencing for retinoblastoma demonstrated improved sensitivity to somatic SVs, particularly insertions, relative to short-reads, as well as highlighting the ability of long reads to phase variants and show both copies of the gene are impacted (Zheng et al. 2024a). In addition, long-read phasing and methylation information can be used to distinguish the tumor clones and haplotypes (Ermini and Driguez 2024; Fu et al. 2024; Simpson 2024). A few methods for multiallelic phasing exist, such as WhatsHap for polyploid phasing (Schrinner et al. 2020) or Strainy for bacterial strain phasing (Table 1; Kazantseva et al. 2024). However, specialized methods for phasing multiple cancer clones are yet to be developed.

De novo assembly, showcased by many recent germline long-read studies, could also reveal somatic variation in cryptic and difficult-to-map regions of tumor genomes (Garg 2023; Ijaz et al. 2024). It is, however, complicated by tumor heterogeneity, allelic imbalance, and long near-perfect duplications. Some of these challenges can be addressed by the local assembly of tumor-specific regions (Le et al. 2024). Alternatively, high-quality assemblies of matching normal genomes (Xiao et al. 2022) or improved human reference assemblies (Paulin et al. 2025) can enhance the detection of somatic variants. Related approaches that analyze genome graphs can also resolve ecDNA amplicons, which may consist of repeated chromosomal fragments (Giurgiu et al. 2024; Zhu et al. 2024).

### Additional long-range technologies for use in cancer genomics

To further elucidate large, repetitive, and complex genomic regions, such as those surrounding the *MYC* gene—frequently rearranged and amplified on a megabase scale in cancer—long-range technologies have been integrated. Hi-C, a genome-wide chromosome conformation capture technique, identifies large-scale rearrangements by capturing the spatial proximity of genomic regions, and has been widely used for variant phasing and genome assembly (Liao et al. 2023). A recent study used a combination of long-read and Hi-C sequencing to reveal chromosome-scale structure of germline-rearranged genomes (Schöpflin et al. 2022). In another study, chromothripsis-affected chromosomes of esophageal adenocarcinomas were haplotype-resolved and assembled using Hi-C (Ijaz et al. 2024). Further, Hi-C can be used to capture large structural rearrangements and reconstruct complete cancer karyotypes (Brunette et al. 2024), as well as validate the rearranged structure of ecDNA amplicons (Helmsauer et al. 2020). Furthermore, combination of long reads and Pore-C sequencing, which is a Hi-C-like assay but uses long-read sequencing to potentially capture several physically localized segments of DNA simultaneously, generated near-complete T2T assemblies of the human genome, resolving remaining difficult regions and providing a precise, highly continuous framework for structural genomic studies (Koren et al. 2024). Pore-C is particularly useful in studying chromothripsis; long reads allow for better resolution of these complex events by covering repetitive regions and capturing structural variations without amplification (Ulahannan et al. 2019). Combining long reads and Pore-C is particularly useful in ecDNA detection as well as detecting translocations without the need for breakpoint-spanning reads (Hickey et al. 2024).

Another long-range technology is optical genome mapping (OGM), such as Bionano (global change) Genomics's technology. It visualizes sequence motifs on DNA molecules longer than 100 kb, to enable the detection of copy number alterations and structural variations, including chromothripsis and large inter and intrachromosomal translocations. By using fluorescent labels and restriction enzymes, OGM produces long-range genomic maps and sensitively reveals structural variants >500 bp even at low allele frequencies. While OGM does not resolve the specific sequence of a genome, it can be faster and more cost-effective than other methods (karyotyping, copy number variation (CNV) microarrays, polymerase chain reaction (PCR), and/or next-generation sequencing (NGS)) for detecting SVs, with turnaround time in 3–5 days (Levy et al. 2023). Comparison of OGM in 10 different solid tumor types showed it can be used to detect complex SVs including inversions and translocations genome-wide, requiring only as low as 6 µg of input tissue samples (Goldrich et al. 2021). A more specific study comparing optical maps of breast cancer cell line SKBR3 with long-read whole-genome sequencing (LR-WGS) found that 74% of insertions and 80% of deletions detected with Bionano can be confirmed with PacBio and ONT, but lower concordance for inversions and duplications (Savara et al. 2021). Similarly, OGM detected 77.1% of large variants not identified by WGS in cases of lung squamous cell carcinoma, including multiple variants private to primary tumor tissue (Peng et al. 2020).

Beyond its use with genome assembly or resequencing, a unique capability of nanopore sequencing is “adaptive sampling” (or “ReadUntil” sequencing) where the decision to sequence a molecule of DNA or RNA can be determined in real time. This operates by sequencing the first few hundred nucleotides of a molecule (∼1 sec worth of sequencing), and ejecting molecules not of interest. This enables purely computational targeting sequencing from native DNA, enabling the detection of genetic and epigenetic changes, demonstrated in an initial study of 148 genes associated with hereditary cancer (Kovaka et al. 2021).

### New benchmarking standards for cancer genomics

Multiple recent studies have produced long-read sequencing of tumor cell lines and normal cell lines from the same individual with accompanying somatic variant benchmarks (Craig et al. 2016; Fang et al. 2021; Espejo Valle-Inclan et al. 2022; Talsania et al. 2022; Keskus et al. 2024; Liu et al. 2024; Masood et al. 2024); however, the long-read data are typically from older, noisier chemistries, and the amount of high-quality data and curated variant calls is still scarce compared to long-read germline projects. In addition, tumor cell lines can have unstable genomes and/or changes in clonality (Craig et al. 2016; Paulin et al. 2025), requiring characterization of large batches of cells to enable robust benchmarking, similar to the National Institute of Standards and Technology's (NIST's) human genome reference materials for normal cell lines (Zook et al. 2016). Another challenge is a lack of explicit consent to publicly share genomic data for most cancer cell lines.

To address these limitations, the Genome in a Bottle consortium has published extensive short- and long-read sequencing data of a pancreatic tumor cell line and paired normal tissues from a patient that was explicitly consented for public sharing of genomic data and cell lines (McDaniel et al. 2024). Benchmark somatic variants for these samples and additional benchmarks for a diverse set of broadly consented cancer and normal cell line pairs with different somatic mutation signatures are in development to enable the community to develop, optimize, and demonstrate the performance of new sequencing and analysis methods.

## Multiomics with long reads

### Transcriptomics with long reads

Transcriptome analysis facilitates the interpretation of gene expression within dynamic cancer and normal cells at a molecular level, which is crucial for advancing precision oncology (Supplitt et al. 2021). While short-read RNA sequencing has been widely employed in transcriptomic research, it struggles to capture full-length transcripts and complex transcriptional events, particularly alternative splicing (AS) and gene fusions (Byrne et al. 2019). Long-read sequencing technologies overcome these limitations by enabling the sequencing of entire transcripts and revealing the exact sequence and structure of fusion transcripts, thereby providing deeper insights into transcriptome isoform diversity. PacBio uses its SMRT sequencing technology for full-length cDNA molecules, capturing entire transcript isoforms with high accuracy (Logsdon et al. 2020). Comparatively, ONT can sequence both native RNA and full-length cDNA, allowing for the detection of complete transcript structures (Soneson et al. 2019). For example, within noncancer donor tissues, a recent study analyzed a large human long-read RNA-seq data set using the ONT platform from 88 samples from Genotype-Tissue Expression (GTEx) tissues and cell lines, complementing the short-read GTEx resource (Glinos et al. 2022). Through the long-read analysis, the researchers identified over 70,000 novel transcripts for annotated genes, and validated the protein expression of 10% of novel transcripts.

### Characterizing transcriptomic diversity and fusion genes with long reads

Within cancer genomics, long-read transcriptome sequencing has uncovered a large range of RNA diversity inaccessible to short-reads. In 2021, the first full-length gastric cancer (GC) transcriptome database was built, covering the four major GC molecular subtypes and identifying 60,239 nonredundant transcripts—over 66% of which were novel (Huang et al. 2021). These novel isoforms, often expressed at higher levels and with greater variability, provide additional prognostic insights. Another colorectal cancer study integrated short- and long-read RNA-seq data, revealing over 50,000 (>60%) unannotated transcripts and identifying thousands of prognostically significant AS events (Sun et al. 2023), suggesting the necessity of long-read sequencing in capturing transcriptome complexity in tumors.

The accurate detection of fusion genes is necessary due to their prevalence in tumor tissues and their role as driver mutations in various cancers. Taking advantage of long-read sequencing, researchers have developed computational tools such as FusionSeeker (Chen et al. 2023b) and CTAT-LR-fusion (Qin et al. 2025) to promote comprehensive characterization of gene fusions. For example, a study used full-length transcript information to identify a novel three-gene fusion, *BMPR2-TYW5-ALS2CR11*, in a lung cancer cell line, demonstrating the capacity of long-read sequencing to identify complex fusion transcripts beyond mere breakpoint discovery (Davidson et al. 2022). Recently, an analysis of PacBio full-length RNA isoform sequencing data uncovered 23 known and 99 novel fusions in sarcoma samples, including *ASPSCR1-TFE3* fusion, a known marker of sarcomas (Volden et al. 2023).

Specific spliced isoforms play a crucial role in cancer progression, metastasis, and drug resistance, with certain AS events strongly correlated with patient survival (Stricker et al. 2017). In 2022, Veiga et al. (2022) developed a long-read RNA sequencing and annotation platform to predict the functional consequences of spliced isoforms in cancer. Their comprehensive analysis of breast cancer and normal breast samples identified 142,514 isoforms in breast tumors, 66% of which are novel, with many affecting protein-coding exons and potentially altering protein function. In addition, they identified 3059 tumor-specific splicing events, including 35 associated with patient survival and 10 enriched in specific breast cancer subtypes. The findings demonstrate the complexity and clinical significance of these isoforms and splicing events, providing a valuable resource for potential immuno-oncology therapeutic targets.

For more effective application of long-read RNA sequencing in clinical settings, several methods for transcriptome-based identification of disease variation are available. For example, the Capture and Ultradeep Long-Read RNA Sequencing (CAPLRseq) workflow evaluates the impact of various genomic alterations on mRNA structural integrity and expression, including coding and intronic SNVs, as well as structural variants like retrotransposon insertions and large genomic rearrangements (Schwenk et al. 2023). The workflow has been used for diagnosing hereditary nonpolyposis colorectal cancer (HNPCC), also known as Lynch syndrome. In the analysis of 123 hereditary cancer-related transcripts, CAPLRseq reclassified two variants of uncertain significance (VUS) in the *MSH6* and *PMS2* genes as likely pathogenic or benign and confirmed 17 cases of HNPCC/Lynch syndrome by identifying splicing defects and allele-specific expression loss in mismatch repair genes. This highlights the promise of long-read RNA sequencing to improve genetic diagnoses and the interpretation of complex hereditary cancer syndromes.

### Toward long-read single-cell transcriptomics in cancer

Beyond traditional bulk RNA sequencing, long-read single-cell RNA sequencing (LR scRNA-seq) has become increasingly relevant in oncology, providing a more detailed view of the cancer landscape at single-cell resolution. A 2023 ovarian cancer study performed LR scRNA-seq on clinical samples from three patients, producing the deepest PacBio scRNA-seq data set to date (Dondi et al. 2023). They identified 152,000 isoforms, with one-third being novel, and uncovered many cell-type-specific isoforms. Notably, isoform-level analysis that accounted for noncoding isoforms demonstrated that protein-coding gene expression had been overestimated by an average of 20%, suggesting the necessity for isoform-specific quantification. Leveraging the advantages of long-read technology, the study provided evidence suggesting that cancer cells may induce epithelial–mesenchymal transition in tumor microenvironment mesothelial cells, and identified gene fusions, such as *IGF2BP2::TESPA1*, previously misclassified as high *TESPA1* expression in short-read data.

Furthermore, genetic and transcriptomic variations could cause cancer clonal heterogeneity and impact treatment outcomes. Linking genetic to transcriptomic variations is crucial for unraveling the mechanisms underlying treatment resistance in cancer (Vasan et al. 2019). LR scRNA-seq enables simultaneous detection of both genetic and transcriptomic variations. Leveraging this potential, LongSom, a computational workflow for LR scRNA-seq data, was developed to detect de novo somatic SNVs, copy number alterations, and gene fusions, further reconstructing the tumor clonal heterogeneity (Dondi et al. 2025). Application of LongSom to ovarian cancer samples identified clinically relevant somatic SNVs that were missed by short-read scRNA-seq. By integrating somatic SNVs and fusions, LongSom distinguished subclones with varying predicted treatment responses. The work demonstrates LR scRNA-seq is effective for exploring cancer evolution, clonal heterogeneity, and treatment outcome prediction.

## DNA and RNA methylation analysis

### Long-read methylation profiling in cancer

Abnormal DNA methylation, a hallmark of cancer development, typically manifests as genome-wide hypomethylation and site-specific CpG island promoter hypermethylation (Sonar et al. 2024). In cancer cells, hypermethylation drives malignant transformation through silencing tumor suppressor genes and disrupting essential cellular processes, such as cell–cell adhesion and apoptotic pathways. Meanwhile, global hypomethylation leads to genomic instability, activation of transposable elements, and potential oncogene upregulation (Magi et al. 2023b). Therefore, DNA methylation patterns serve as valuable biomarkers for cancer diagnosis, prognosis assessment, and therapeutic decision-making (Jones et al. 2016). Long-read sequencing technologies have enhanced epigenetic profiling by reducing GC bias, identifying CpG islands at lower read depths, and enabling direct examination of native DNA modifications (Ermini and Driguez 2024). ONT detects DNA modifications by measuring changes in the electrical signal as native DNA passes through the nanopore, while PacBio uses real-time kinetic analysis of DNA polymerase activity to infer modifications (Xu and Seki 2020). Importantly, long reads enable methylation phasing alongside genetic variations, revealing ASM patterns associated with complex diseases and cancers, which are challenging with short-read data (Gigante et al. 2019).

Recent studies have demonstrated the power of long-read sequencing in characterizing cancer methylation patterns. Using the ONT PromethION platform, Ewing et al. (2020) accurately assessed transposable element methylation in hepatocellular carcinoma (HCC) tissues, detecting pronounced demethylation of LINE-1 retrotransposons in cancer. Later, nanoEM successfully analyzed two tandem LINE-1 elements within *HECTD2* introns that traditional short-read methods like EMseq and whole-genome bisulfite sequencing (WGBS) failed to fully capture (Fig. 2E; Sakamoto et al. 2021a). Analysis of the Personalized OncoGenomics data set revealed that long-range phasing enables identification of allelically differentially methylated regions (aDMRs) in cancer genes, such as *RET* and *CDKN2A*. This study also observed *MLH1* promoter hypermethylation in Lynch syndrome, and showed promoter methylation in *BRCA1* and *RAD51C* as potential drivers of homologous recombination deficiency in cancers lacking known mutations (O'Neill et al. 2024). A medulloblastoma study using long-read sequencing revealed ASM effects, complex rearrangements with differential methylation, and distinct methylation patterns in cancer driver genes (Rausch et al. 2023). Besides, ONT-based methylation profiling enables rapid central nervous system (CNS) tumor subtype classification (Patel et al. 2022), which could be performed using real-time sequencing during surgery (Vermeulen et al. 2023). Similarly, PacBio sequencing, integrated with advanced computational methods, has shown superior performance in detecting distinctive methylation patterns with greater accuracy than short-read technologies, offering enhanced diagnostic power in cancer studies (Choy et al. 2022; Ni et al. 2023)

Assessing cfDNA methylation in plasma holds great promise for the early, noninvasive cancer detection (Li and Zhou 2020). Traditional bisulfite or enzymatic conversion methods introduce biases, which are further complicated by limited cfDNA yields from plasma samples, making comprehensive methylome characterization challenging. Long-read sequencing technology enables direct, unbiased methylation identification, advancing liquid biopsy applications (Lau et al. 2023). Notably, the longer reads provide more CpG sites, enriching methylation information in plasma DNA. These extended methylation patterns serve as more complete “molecular barcodes” compared to shorter fragments, improving tissue-of-origin analysis specificity and supporting the development of crucial noninvasive cancer biomarkers (Choy et al. 2022). In 2022, an innovative methylation analysis used nanopore sequencing and detected cell-of-origin and cancer-specific methylation, suggesting a liquid biopsy approach would be possible with long reads (Katsman et al. 2022). Later, Lau et al. (2023) developed a single-molecule sequencing strategy to analyze cfDNA methylomes, and observed distinct methylation patterns between cancer patients and healthy individuals. By comparing the methylomes of matched tumors and immune cells, they characterized cfDNA methylation in cancer patients for longitudinal monitoring throughout treatment. Finally, an HCC study using PacBio sequencing discovered previously unreported long cfDNA fragments (>1 kb) in cancer patients. They designed the “HCC Methylation Score,” a metric reflecting single-molecule methylation patterns associated with cancer, and found that long cfDNA improved discriminatory power than short cfDNA (Choy et al. 2022).

Long-read sequencing technologies have also advanced the study of RNA modifications, particularly *N*^6^-methyladenosine (m6A) patterns. m6A influences multiple aspects of RNA metabolism, such as translation, degradation, splicing, and export, playing crucial roles in tumor regulation and development (Wang et al. 2022). ONT detects RNA modifications by sequencing native RNA molecules directly and capturing the changes in electrical signals, while PacBio indirectly infers modifications from cDNA sequencing (Xu and Seki 2020). Using native RNA sequencing, Jenjaroenpun et al. (2021) identified cell-type-specific m6A patterns in lung cancer cell line, discovering distinct modifications absent in reference cells. More recently, a liver cancer study identified 3968 potential m6A modification sites across 1396 genes and revealed these m6A-modified genes involved in multiple key cellular processes (Arzumanian et al. 2023). A recent long-read study of clear cell renal cell carcinoma (ccRCC) using nanopore identified 9644 genes with altered m6A modifications, with 5224 genes showing concurrent changes in both m6A modification and RNA expression (Li et al. 2024). Notably, this analysis found four obesity-associated genes with prognostic significance in ccRCC. These studies demonstrate the potential of long-read sequencing to detect RNA base modifications, providing valuable insights into the cancer epitranscriptome and the exploration of novel therapeutic targets.

### Methylation detection, analysis, and visualization using long reads

Recent advances in computational methods have improved methylation detection from long-read sequencing data. The newest ONT basecallers enable direct detection of 5mC and 5hmC from single DNA reads (Liu et al. 2021). Advanced tools employ pretrained models, like hidden Markov models and deep neural networks, to translate electrical current signals into specific bases (https://github.com/nanoporetech/megalodon, Ni et al. 2019). The recently developed Uncalled4 further improves the performance in detecting DNA and RNA modifications, identifying 26% more m6A modifications than Nanopolish using m6Anet in seven normal and cancerous human cell lines, including in cancer-implicated genes like *ABL1* and *JUN* (Table 1; Fig. 2F; Kovaka et al. 2024). Similar to how accurate read alignments are needed for robust detection of sequence variations, Uncalled4's improved signal alignments lead to improved detection of epigenetic changes. Using PacBio sequencing data, Tse et al. (2021) developed a convolutional neural network method named the holistic kinetic model for genome-wide 5mCpG detection. To mitigate high subread depth requirements, PacBio later introduced primrose that can achieve 85% read-level accuracy in 5mCpG detection (Ni et al. 2023). Relatedly, ccsmeth, a deep learning method for PacBio data, demonstrates 90% detection accuracy for DNA 5mCpGs at single-molecule resolution, facilitating precise, genome-wide methylation profiling (Table 1; Ni et al. 2023).

ASM, occurring throughout the human genome with frequent presence at imprinted loci, influences gene regulation and disease susceptibility (Benton et al. 2019). To identify ASM using long reads, NanoMethPhase (Akbari et al. 2021) integrates SNV and methylation signals from nanopore, showing successful ASM detection from about 10× coverage in the COLO829BL B lymphoblast cell line (Table 1). MethPhaser enhances phasing accuracy by connecting phase blocks through heterozygous methylation information, particularly improving resolution across human leukocyte antigen (HLA) and other medically relevant genes (Table 1; Fu et al. 2024). The ccsmethphase Nextflow pipeline, designed for PacBio data, enables accurate genome-wide ASM identification, even in repetitive regions (Table 1; Ni et al. 2023). Differential methylation analysis has further expanded our understanding of cancer epigenetics. PoreMeth provides high-resolution detection of DNA methylation alterations between sample pairs for both CpG islands and sparse CpG regions (Magi et al. 2023b). Its application in acute myeloid leukemia (AML) revealed drug resistance phenotypes are driven by selective epigenetic alterations in transcription factor regions. xPore enables differential analysis from direct RNA sequencing data. It identified between 800 and 2000 differentially modified sites per cancer cell line when comparing five cancer cell lines against HEK293T-KO cells, with most sites conforming to the m6A DRACH motif (Pratanwanich et al. 2021).

Efficient visualization tools promote the analysis and interpretation of methylation patterns. NanoMethViz provides a comprehensive solution for methylation data visualization by processing outputs from diverse methylation callers and implementing efficient data compression for large-scale data sets (Su et al. 2021). Its multiresolution visualization capabilities allow researchers to explore methylation patterns from broad genomic regions down to single-read resolution at specific loci of interest. Recent standardization efforts by the Global Alliance for Genomics and Health (GA4GH) have introduced two new tags (MM and ML) to the SAM/BAM file specification. Using this standardized format of files, modbamtools enables visualization, manipulation, and comparative analysis of base modifications with robust performance (Razaghi et al. 2022). The tool produces publication-quality visualizations and supports downstream analyses of methylation patterns. Additionally, WashU Epigenome Browser has added the modbed track, which displays modification details at single-read and aggregated levels across multiple resolutions. Users can access these visualizations by uploading modbed files locally or through URLs on the WashU platform (Li et al. 2023a,b).

### Chromatin accessibility and conformation

Histone modifications influence chromatin structure and gene regulation in carcinogenesis (Yang et al. 2022). For example, alterations in histone-modifying enzymes, such as *SETD2* mutations in renal cell carcinoma, lead to dysregulated gene expression (Yu et al. 2023). Chromatin conformation capture technologies, such as Hi-C or Pore-C were originally developed to study the 3D organization of the genome (Belton et al. 2012). Somatic SVs can change the spatial organization of the genome, which may be specific to different cancer types (Dubois et al. 2022). For identifying mechanisms of cancer development, Hi-C helped identify *TP53* acting as a regulator of chromatin structure (Serra et al. 2024) as well as how *TP53* loss causes whole-genome doubling that impacts chromatin segregation (Lambuta et al. 2023). A related technique, scNanoHi-C (Li et al. 2023b), is a single-cell long-read concatemer sequencing method to reveal high-order chromatin structures within individual cells. Their results suggest that extensive high-order chromatin structures exist in active chromatin regions across the genome, and multiway interactions between enhancers and their target promoters were systematically identified within individual cells.

A related technique called Fiber-seq leverages the ability of long-read sequencing to measure chromatin accessibility (Stergachis et al. 2020). This process operates by “stenciling” the structure of individual chromatin fibers onto their composite DNA templates using nonspecific DNA *N*^6^-adenine methyltransferases. Single-molecule long-read sequencing of chromatin stencils then enables nucleotide-resolution readout of the primary architecture of multikilobase chromatin fibers. Fiber-seq exposed widespread plasticity in the linear organization of individual chromatin fibers and illuminated principles guiding regulatory DNA actuation, the coordinated actuation of neighboring regulatory elements, single-molecule nucleosome positioning, and single-molecule transcription factor occupancy.

## Future opportunities and challenges in long-read cancer genomics

### Scaling to large samples from diverse populations

**Figure 3.**
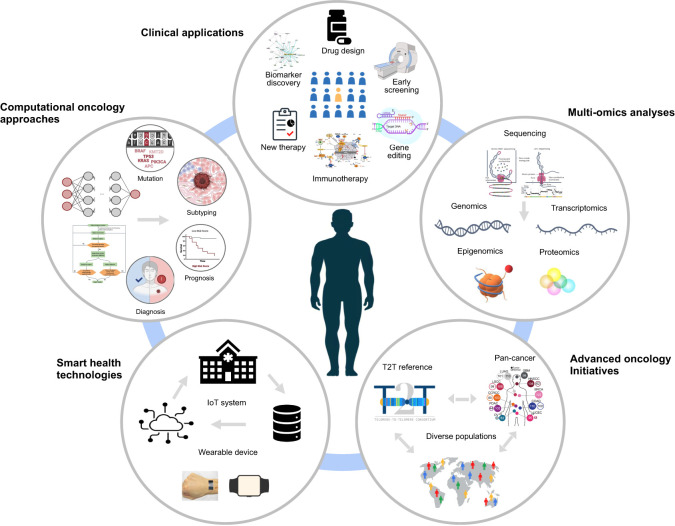
Advanced strategies in precision oncology. (*Top right* panel) Multiomics analyses illustrate the integration of advanced sequencing technologies with various omics approaches to elucidate the molecular foundations of cancer pathogenesis and progression. The sequencing, genomics, and transcriptomics illustrations are from Kovaka et al. (2023), the epigenetics illustration is adapted from Mehrmohamadi et al. (2021), and the proteomics illustration is from Zhang et al. (2021). (*Bottom right* panel) Advanced oncology initiatives highlight emerging trends such as T2T reference genomes and pan-cancer studies across diverse populations. The pan-cancer illustration was adapted from Savage et al. (2024). (*Top left* panel) Computational oncology approaches demonstrate the application of advanced algorithms and artificial intelligence methods in cancer research. Four potential applications and a deep learning model adapted from Unger and Kather (2024), along with a computational model flowchart from Ma and Gurkan-Cavusoglu (2024) are depicted. (*Bottom left* panel) Smart health technologies show the development of applications for efficient data collection, transmission, real-time monitoring, and treatment in cancer care, such as internet of things (IoT) systems and wearable devices. The wearable device illustration on the *left* is modified from Ma et al. (2023). (*Top center* panel) Clinical applications illustrate some key clinical directions in precision oncology. The biomarker discovery illustration is modified from Kawata-Shimamura et al. (2022). The early screening illustration is from Liao et al. (2022), the gene editing illustration from Zhang et al. (2021), and the immunotherapy illustration from Guerrouahen et al. (2020).

The rapidly evolving landscape of cancer genomics and precision oncology necessitates accurate and comprehensive variation profiling across large, diverse populations. Long-read sequencing has emerged as an indispensable tool for cancer studies, but it requires suitable HMW DNA extraction protocols and size selection methods for optimal performance (Ermini and Driguez 2024). However, HMW DNA molecules are often challenging to obtain from clinical tissues compared to the input for short-read reactions. A comparative study demonstrated that cryopreserved tumor samples provide superior DNA quality, integrity, and quantity compared to FFPE tissues (Okojie et al. 2024). High-quality material benefits long-read sequencing, particularly for detecting large SVs such as chromosomal translocations, inversions, and copy number variations, common in genomically unstable tumors. The prevalent use of FFPE tissue may compromise the identification of these critical variations, underscoring the need to optimize sample selection, preservation, and DNA extraction methods (Haile et al. 2019). Relatedly, high-depth sequencing is needed to detect low allele fraction variants, especially when considering tumor heterogeneity and mixtures of tumor and normal cells (Talsania et al. 2022). It is also more costly, and thus guidelines for the depth of sequencing for clinical sequencing need to be established (Amarasinghe et al. 2020). Besides, long-read sequencing technologies face limitations in accurately identifying certain classes of small variants, especially indels, in homopolymeric and low-complexity regions (Nyaga et al. 2024). Although ONT's new R10 pore provides better resolution in homopolymer regions, systematic biases persist, particularly with certain *k*-mers varying in the distinctness of the signals they produce (Amarasinghe et al. 2020). Similarly, PacBio reads, despite their high overall accuracy, exhibit bias in homopolymer regions (Fig. 3; Amarasinghe et al. 2020). Addressing these challenges will require continued advancement in both sequencing chemistry and computational algorithms.

Another major consideration is that researchers are increasingly recognizing the importance of including diverse populations in oncology clinical trials, aiming to uncover critical disparities in cancer incidence, prevalence, and treatment outcomes (Fig. 3; Vidal et al. 2024). For instance, a study on nonsmall-cell lung cancer revealed significant differences in survival rates and *EGFR* mutation frequencies between Asian and non-Asian patients treated with gefitinib (Mok et al. 2018). Moving beyond single-cancer studies, pan-cancer projects have shed light on genomic differences across various cancer types (Fig. 3). A recent analysis of 7152 tumors sequenced with short-reads uncovered metastatic tumors typically exhibit lower intratumor heterogeneity, higher genomic instability, and more frequent SVs compared to primary tumors (Martínez-Jiménez et al. 2023). As long-read sequencing technologies improve and become more cost-effective, their applications in cross-population and pan-cancer studies promise to reduce genetic biases in complex genomic regions and elucidate the shared genetic basis across cancers, thereby revealing the global landscape of human cancer genetics.

Considering current pan-cancer studies mainly use short-read sequencing, we advocate for the utilization of long-read sequencing technologies to uncover previously missed variant characteristics and establish stronger links between variations, cancer driver genes, and tumor progression. Furthermore, multiple studies have verified the potential of T2T-CHM13 reference genome in cancer research for improved variant detection accuracy (Paulin et al. 2025). As sequencing technology advances and becomes more accessible, the possibility of complete personalized T2T genome assemblies, with both parental haplotypes phased from telomere to telomere, emerges as a potential new standard in human genetics (Fig. 3; Miga and Eichler 2023). We expect this effort would pave the way for deeper exploration of variation mechanisms and their impact on human health. Overall, expanding pan-cancer studies to encompass large-scale, high-quality sequencing data from diverse populations promise to enhance knowledge of tumor biology and accelerate the development of personalized, more effective cancer treatments in the future.

### Long-read data analysis challenges

Long-read sequencing introduces several unique computational challenges over standard short-read approaches (Fig. 3). Firstly, computational requirements are often higher for base-calling raw data from long-read sequencing, such as with PacBio HiFi sequencing and Oxford Nanopore (Wenger et al. 2019; Baid et al. 2022; Pagès-Gallego and de Ridder 2023). Furthermore, sequencing chemistries and base-calling algorithms have been evolving rapidly, posing challenges in validating pipelines and requiring extensive frequent benchmarking. Relatedly, long reads can have unique computer hardware requirements. For example, the algorithm advances related to genome sketching (Rowe 2019) allow for performant alignment of long reads given sufficient I/O bandwidth, along with the incorporation of deep learning architectures such as Transformers (Vaswani et al. 2017) for DeepConsensus that heavily use graphics processing units (GPUs) to process reads. The large data can make data security requirements for patient genomic data more challenging, though risk assessment and management exercises (Pulivarti 2023) can yield appropriate analysis and storage infrastructure including the use of cloud platforms that meet NIH Genomic Data Sharing (GDS) policy (https://datascience.cancer.gov/data-commons/cloud-resources, https://sharing.nih.gov/genomic-data-sharing-policy).

SVs are key drivers of cancer development, underpinning major driver mutations and somatic copy number alterations (Elrick et al. 2024). Copy number alterations (CNAs), which include large deletions and amplifications spanning genes or entire chromosomes, influence cancer progression by modulating oncogene and tumor suppressor gene expression (Muñoz-Barrera et al. 2022). While long-read sequencing has enhanced our ability to detect these variations, the effective representation and comparison of complex SVs and CNAs require new standards and tools. The distinction between SVs and CNAs often blurs due to the lack of universal size thresholds for classification. In general, SVs tend to have precise breakpoints, whereas CNAs have fuzzy boundaries due to the coverage-based detection methods. In some cases, the breakpoints of a CNA may be caused by a translocation, which is generally called an SV, but there are no standards for how to represent this relationship. New tools are needed to compare somatic SV and CNA callsets both for benchmarking and cross-individual comparisons. While more robust comparison tools for sequence-resolved SVs have recently been developed for sequence-resolved germline variants (English et al. 2022; Dunn et al. 2024), these tools do not generally accommodate somatic SVs and CNAs, which often are much larger and sometimes more complex with many breakpoints.

Beyond these considerations, data interpretation and visualization is a critical component of analyzing the complex somatic structural variation that can be discovered by long reads in cancer genomes. An array of existing tools enable data overview and summarization, sense-making, and hypothesis generation with more improvements continually being developed. Considering the canonical visualization approach of overview first, zoom and filter, then details on demand (Shneiderman 1996), Genome Ribbon (Nattestad et al. 2021), and PGR-TK (Chin et al. 2023) provide a mix of chromosome-scale views paired with read-level or gene data.

### Application of deep learning in cancer research

Genomic alterations drive the development and progression of cancer, reshaping the genetic landscape as the disease advances. To address challenges in cancer genomics, such as sequencing artifacts, tumor-normal cross-contamination, and low-frequency somatic mutations, several artificial intelligence (AI) models have been developed for long reads to improve base-calling accuracy (Amarasinghe et al. 2020; Pagès-Gallego and de Ridder 2023) and enhance the variation detection (Fig. 3; Zheng et al. 2023; Park et al. 2024; Wang et al. 2024). As long-read sequencing enables more accurate identification of structural variations, distinguishing driver from passenger SVs has become crucial in cancer genome analysis. Murakami et al. (2024) developed an explainable artificial intelligence (XAI) framework that integrates knowledge graphs and deep tensors with large language models to predict pathogenicity and elucidate mechanisms of SVs involving fusion genes. Beyond SV identification and interpretation, accurate tumor characterization benefits to diagnosis, prognosis, and treatment selection (Singh et al. 2021). The Mutation-Attention (MuAt) deep neural network was developed to learn representations of diverse genetic alterations, integrating SNVs/multiple nucleotide variants (MNVs), indels, and SV breakpoints through multimodal data embeddings (Sanjaya et al. 2023). This approach enables precise identification of histological tumor types and specific entities, such as *SHH*-activated medulloblastoma and *SPOP*-associated prostate cancer, advancing the potential for precision oncology. To understand the complex nature of tumor pathogenesis, researchers have developed multiomics data fusion algorithms (Chaudhary et al. 2018; Picard et al. 2021) that integrate diverse molecular profiles, providing a more detailed view of cancer development. A recent Tumor MultiOmics pretrained Network (TMO-Net) model (Wang et al. 2024), integrates multiomics data from 32 cancer types, incorporating genomic, transcriptomic, proteomic, and metabolomic information. TMO-Net demonstrates enhanced performance in various oncology tasks, including gene mutation prediction, cancer subtype classification, drug response forecasting, and prognosis prediction. Beyond omics applications, AI has been widely adopted to advance cancer research in various fields, including biomedical image analysis, novel drug candidate identification, and the prediction of drug–drug interactions (Li et al. 2023a; Han and Tao 2024). These ongoing efforts have accelerated the drug discovery process and opened new avenues for personalized cancer treatments.

### Translating long-read research into the clinic

Long-read sequencing holds immense potential for transforming cancer diagnostics and treatment. However, transitioning this technology from a powerful research tool to a routine clinical application presents several challenges, alongside promising opportunities.

One of the primary obstacles to the clinical adoption of long-read sequencing is data accessibility, especially the cost, throughput, and automation of the technology. Although technologies like Oxford Nanopore, PacBio, and potentially other long-read platforms (Zhang et al. 2024) offer unmatched resolution of structural variants and complex genomic regions, they remain more expensive compared to short-read sequencing. For widespread clinical use, it is essential to reduce these costs and increase throughput to make long-read sequencing a viable option for routine diagnostics. This is particularly important for somatic mutations because high coverage is required to detect low-frequency mutations. Relatedly, the clinical environment demands consistent and reproducible results, necessitating highly automated and standardized workflows. Presently, long-read sequencing involves multiple manual steps—from DNA extraction to data analysis—that are rapidly improving, introducing variability, and extending turnaround times. To facilitate clinical adoption, the development of fully automated sequencing workflows, including robust and stable software pipelines, is crucial (Fig. 3). These systems must be capable of efficiently processing and interpreting the vast data sets generated by long-read sequencing. Ensuring that the technology meets clinical standards for accuracy, reproducibility, and patient safety is critical.

After the experimental requirements for long-read sequencing, the largest remaining challenge is in the interpretation of the variations found. Long-read sequencing frequently uncovers a multitude of variants, including many that are of unknown significance (VUS). This presents a challenge for clinicians in making informed decisions based on these results. Developing comprehensive databases and catalogs of variants best resolved by long reads, supported by initiatives like *All of Us*, ColorsDB, and the 1000 Genomes Project, as well as advanced interpretation algorithms like CADD-SV and Watershed-SV, are essential to help clinicians accurately interpret these findings. Additionally, integrating data from these initiatives can enhance our understanding of variants across diverse populations.

Despite these challenges, we are optimistic for the growing adoption of long reads for cancer research and clinical care. Long-read sequencing excels at detecting complex genomic, transcriptomic, and epigenomic variants, including structural variants, gene fusions, and TRs, which are often missed by short-read technologies. This capability opens new avenues for identifying clinically relevant variants that could serve as biomarkers for cancer diagnosis, prognosis, and treatment selection (Fig. 3). For example, known fusions that are difficult to detect with short-reads can be more easily identified with long reads, offering crucial information for targeted therapies.

One promising opportunity to increase the accessibility of long reads is the development of targeted long-read sequencing panels focusing on specific genes or genomic regions known to be implicated in certain cancers (Iyer et al. 2024). These panels could be particularly valuable for genes located in segmental duplications or other challenging regions poorly resolved by short-reads. Such targeted panels would provide actionable insights, facilitating personalized medicine, and enabling the routine clinical use of long-read sequencing. Another opportunity is to design and conduct meaningful studies with smaller cohorts, such as family-based studies. These studies can provide valuable insights into inherited cancer susceptibility and the role of rare variants in disease. In particular, trio or quad family structures help phase variants to trace cancer predisposition alleles within families, while facilitating the filtration of background variations unlikely to contribute to cancer risk.

As more success stories emerge from programs like Genomics England Cancer 2.0 (https://www.genomicsengland.co.uk/initiatives/cancer), the Personalized OncoGenomics program (O'Neill et al. 2024), and the programs at Children's Mercy Hospital, where long-read sequencing has led to significant clinical breakthroughs, the technology will gain greater acceptance in the clinical community. Demonstrating the real-world impact of long-read sequencing on patient outcomes will be key to driving its adoption in routine clinical practice. While there are remaining challenges to overcome, the opportunities presented by long-read sequencing in the clinical setting are substantial. By addressing cost, automation, sample requirements, and variant interpretation, and by building on successful case studies, long-read sequencing has the potential to revolutionize cancer diagnostics and personalized medicine.
